# Mastering particle size analysis: lessons, challenges, and future directions from the FDA–CRCG workshop

**DOI:** 10.1186/s41120-026-00148-4

**Published:** 2026-04-06

**Authors:** Xiaoming Xu, William C. Smith, James E. Polli, Christine L. Plavchak, Bin Qin, Yan Wang, Nicholas Holtgrewe, Sam G. Raney, Andre O’Reilly Beringhs, Yihua Bruce Yu, Marc Taraban, Vishalakshi Krishnan, Anna Schwendeman, Dana C. Hammell, Jeffrey D. Clogston, Alan F. Rawle, Jernej Grmaš, Yousif Ayoub, Bernard Domnic, Rama Subba Reddy, Joseph Duncan, Matthew McGann, Jeff Bodycomb, Carl C. L. Schuurmans, Michiel Hermes, Jonathan G. Mehtala, Prashun Roy, Kevin Lance, Collin Britten, Kiwan Park, Daniel Beach, Matthew Elmer, Keith B. Rodenhausen, Zhen Xu, Haiou Qu, Kai-Wei Wu, Maxwell Korang-Yeboah, Yuan Zou, Ji Li, Alaa H. Abuznait, Colette Quinn, Anabelle Ethier, Kendice Ip, Dinesh Dhamecha, Qi Li, Dongkai Zhu, Rutu Valapil

**Affiliations:** 1 Office of Pharmaceutical Quality, Center for Drug Evaluation and Research, U.S. Food and Drug Administration, Silver Spring, MD 20993 USA; 2 Department of Pharmaceutical Sciences, School of Pharmacy, University of Maryland, Baltimore, MD 21201 USA; 3 Office of Generic Drugs, Center for Drug Evaluation and Research, U.S. Food and Drug Administration, Silver Spring, MD 20993 USA; 4 Department of Pharmaceutical Sciences, College of Pharmacy, and the Biointerfaces Institute, University of Michigan, Ann Arbor, MI 48109 USA; 5 Nanotechnology Characterization Laboratory, Cancer Research Technology Program, Frederick National Laboratory for Cancer Research, Frederick, MD USA; 6 Hardwick, MA US; 7 Sandoz, Lek d.d., Verovškova Ulica 57, Ljubljana, SI – 1526 Slovenia; 8 Teva Pharmaceuticals, 18 Eli Hurvitz St., Kfar Saba, 4450029 Israel; 9 Teva Pharmaceuticals, 400 Interpace Parkway, Bldg A, Parsippany, NJ 07054 USA; 10 Dr. Reddy’s Laboratories, Ltd (IPDO), Bachupally, Medchal-Malkajgiri District, Telangana, 500090 India; 11 Viatris, 3711 Collins Ferry Road, Morgantown, WV 26505 USA; 12 Bettersize Inc., 3185 Airway Avenue Ste C-2, Costa Mesa, CA 92626 USA; 13 HORIBA, 20 Knightsbridge Road, Piscataway, NJ 08854 USA; 14 InProcess-LSP, Kloosterstraat 9, Oss, 5349AB The Netherlands; 15 Malvern Panalytical Inc., 2400 Computer Dr, Suite 2100, Westborough, MA 01581 USA; 16 Microtrac MRB, Part of Verder, 11 Penns Trails #300, Newtown, PA 18940 USA; 17 Unchained Labs, 4747 Willow Rd, Pleasanton, CA 94588 USA; 18 Waters/Wyatt Technology, 6330 Hollister Ave, Santa Barbara, CA 93117 USA; 19 Bettersize Inc., 3185 Airway Ave C2, Costa Mesa, CA 92626 USA; 20 Anton Paar USA, Inc., 10215 Timber Ridge Drive, Ashland, VA 23005 USA; 21 Sterinova, 3005 Ave, José-Maria-Rosell, St-Hyacinthe, Qc J2S 0J9 Canada; 22 PCCA, 9901 South Wilcrest Dr., Houston, TX 77099 USA

**Keywords:** Particle size distribution, Dynamic light scattering, Laser diffraction, Complex drug products, Colloidal dispersion, Suspension, Measurement robustness, Inter-laboratory comparability, Regulatory science, FDA, Center for Research on Complex Generics (CRCG) workshop

## Abstract

Particle-size distribution (PSD) measurement is a cornerstone of pharmaceutical development and regulatory assessment, influencing formulation stability, dissolution, product quality, and performance. Yet the seemingly straightforward nature of “size measurements” often conceals substantial scientific and practical complexity. To strengthen shared understanding and advance good measurement practices, the U.S. Food and Drug Administration (FDA) and the Center for Research on Complex Generics (CRCG) co-hosted a two-day hybrid workshop, “Mastering Particle Size Analysis: A Step-by-Step Illustration of Techniques and Best Practices,” held on September 23–24, 2025. The workshop brought together scientists from regulatory agencies, industry, academia, and instrument manufacturers to examine the principles, data interpretation, and analytical procedure robustness of two widely used techniques—Dynamic Light Scattering (DLS) and Laser Diffraction (LD). The program integrated scientific lectures, vendor-led demonstrations, and structured small-group discussions organized around real-world case studies. Prior to the workshop, participating vendors conducted independent characterization of five pre-workshop materials representing diverse formulation types, providing a shared baseline for comparison. In total, 2823 participants registered for the workshop, including 127 for in-person attendance and 2696 for virtual attendance from across the global pharmaceutical community. Of those that registered, 119 participants attended in-person, and 1464 attended virtually during the two days. Discussions throughout the workshop emphasized that PSD data cannot be interpreted in isolation because what is measured depends on the model assumption, sample condition, and analytical purpose. Participants concluded that reliable characterization demands “fit-for-purpose” analytical procedure design, transparent reporting, and a clear linkage between measurements and material function. Major outcomes included (i) recognition that methodological transparency outweighs pursuit of a single “true” particle size; (ii) identification of the dominant sources of inter-laboratory variability; and (iii) broad consensus on the need for harmonized terminology, validation criteria, and reporting expectations. The workshop closed with collective commitments to prepare a community white paper and to initiate an inter-laboratory study aimed at improving comparability of PSD data across techniques, platforms, and laboratories—steps that will help ensure measurement integrity and regulatory confidence in complex drug development.

**Supplementary Information:**

The online version contains supplementary material available at 10.1186/s41120-026-00148-4.

## Introduction

The control of particle size lies at the heart of modern pharmaceutical science, linking formulation design, manufacturing robustness, product performance, and regulatory assessment (Louey et al. 2025). Whether in suspensions, emulsions, or polymeric matrices, the particle-size distribution (PSD) influences virtually every aspect of product quality from dissolution rate and content uniformity to stability and delivery efficiency. Yet despite its ubiquity, particle-size analysis remains one of the most conceptually intricate tasks in drug product characterization. Laboratories routinely perform PSD testing, but what appears to be a straightforward measurement often conceals deep scientific complexity. Instruments may yield precise numerical outputs, yet those numbers depend on model assumptions, sample conditions, and measurement procedure parameters that are not always recognized or reported. As a result, PSD values that seem definitive in isolation can vary widely when different methods or even the same methods are applied to the same sample (Merkus 2009).

These challenges become particularly evident in the analysis of complex drug products such as emulsions, colloidal dispersions, suspensions and polymeric matrices, where particle-size information is not merely descriptive but fundamental to establishing product quality and bioequivalence. For such systems, the physical interpretation of “size” depends on the principle of measurement, whether based on optical scattering, sedimentation, or diffusion, and may therefore represent distinct physical states across techniques. Despite decades of experience and advanced instrumentation, laboratories still report divergent results for identical materials, underscoring the gap between measuring a number and understanding what that number represents. Such inconsistencies have tangible consequences for product development, technology transfer, and regulatory assessment, underscoring the need for greater clarity and scientific dialogue across stakeholder communities.

The opening remarks of the workshop framed this paradox succinctly: *particle-size analysis may appear simple, yet it is deceptively complex.* What analysts report as “size” often reflects other underlying physical properties governed by the measurement principle and experimental conditions. Much like the parable of the blind men and the elephant, analysts may each observe a fragment of a broader truth measured by the technique used on given sample preparation, each perspective valid within its context but incomplete in isolation. The message emphasized that progress in particle-size science requires both humility and persistence: understanding why a measurement is performed and what the data is providing is as essential as mastering how the measurement is carried out (Center for Research on Complex Generics (CRCG) 2025a).

Recognizing the importance of shared understanding, the U.S. Food and Drug Administration (FDA) and the Center for Research on Complex Generics (CRCG) jointly organized a workshop to strengthen the scientific and practical foundations of PSD measurement. The initiative was driven by the recurring observation that successful characterization of complex generics required not only advanced instrumentation but also a common conceptual framework among regulators, industry, and academia. The two-day workshop, titled “Mastering Particle Size Analysis: A Step-by-Step Illustration of Techniques and Best Practices,” aimed to foster that shared perspective by combining lectures, demonstrations, and collaborative exercises designed to bridge scientific principles with what regulatory agencies need to assess the quality of drug products analyzed (Center for Research on Complex Generics (CRCG) 2025b).

The specific objectives were to (1) illustrate how Dynamic Light Scattering (DLS) and Laser Diffraction (LD) techniques are being applied to both straightforward and challenging samples; (2) explore how instrument settings, sample handling, and analytical assumptions affect data quality and comparability; and (3) identify opportunities for harmonization in method development, validation, and reporting. These goals align with FDA’s broader mission to promote measurement transparency and reproducibility in pharmaceutical quality assessment.

Hosted at The Universities at Shady Grove (USG) campus, the workshop adopted a hybrid format that combined in-person and virtual participation. This approach enabled broad engagement from across the global scientific community while maintaining the interactive format essential for hands-on learning. Day 1 focused on DLS, emphasizing applications on nanoparticulate and colloidal systems where Brownian motion and optical scattering dominate. Day 2 centered on LD, addressing micron-scale suspensions and powders through interpretation of angular light-intensity patterns. By pairing these complementary techniques, the workshop spanned the entire particle size spectrum relevant to pharmaceutical products, ranging from tens of nanometers in colloidal systems such as micelles or lipid-based dispersions to micrometer-scale particles in suspensions and powders, and highlighted how apparent simplicity in measurement can conceal the complexity of diverse physical phenomena affecting the outcomes.

The workshop was intentionally designed to progress from conceptual understanding to practical application and collective reflection by a workshop planning committee that included representatives from the FDA, industry, academia, and the CRCG. Each day opened with plenary presentations providing a theoretical and regulatory context, followed by vendor-led demonstrations illustrating how instrument parameters influence results. Structured breakout sessions then allowed participants to discuss real datasets, identify sources of variability, and share strategies for improving reproducibility. The event concluded with a “Waypoint Exercise,” in which participants distilled key lessons and priorities for future collaboration (Food and Drug Administration (FDA), Center for Research on Complex Generics (CRCG) 2025a). This progression—from theory to practice to synthesis—mirrored the workshop’s overarching aim: to advance not just technical capability but also the shared scientific culture underpinning reliable particle-size analysis.

Preceding the event, participating instrument vendors received five pre-workshop materials representing diverse formulation types: cyclosporine emulsion, iron sucrose colloidal dispersion, phytonadione colloidal dispersion, triamcinolone acetonide suspension, and microcrystalline cellulose (MCC). Vendors were asked to measure each sample using their own instruments and document their method-optimization process in detail. These pre-workshop datasets served as common reference points for group analysis and comparison during the sessions, enabling participants to examine how differences in preparation, optical modeling, and interpretation contribute to variability (Food and Drug Administration (FDA), Center for Research on Complex Generics (CRCG) 2025b). Summaries of the vendor data are provided in Appendix A.

The workshop attracted 2823 registrants worldwide, including 127 for in-person attendance and 2696 for virtual attendance from across the global pharmaceutical community. Of those that registered, 119 participants attended in-person, and 1464 attended virtually during the two days. For those who were unable to attend on the days of the workshop, all presentation files and session video recordings were made available on the CRCG website (Center for Research on Complex Generics (CRCG) 2025b). The diverse representation of regulators, industry scientists, academics, and instrument developers ensured a comprehensive exchange of perspectives. The following sections of this paper summarize the plenary presentations, breakout discussions, and collective reflections that captured the workshop’s central scientific messages and practical outcomes.

## Workshop presentations — key messages from day 1 and day 2

### Overview

Each day of the workshop followed a structured sequence of opening remarks, scientific lectures, case studies, and vendor demonstrations, providing participants with a comprehensive view of the current landscape of particle-size analysis. Day 1 focused on DLS—addressing characterization of nanometer-scale and colloidal systems—while Day 2 centered on LD, emphasizing analysis of micron-scale suspensions and powders. Together, the sessions bridged fundamental physics, practical analytical procedure development, and regulatory considerations. The program was deliberately designed to balance theory and practice, illustrating how scientific understanding of foundational principles, analytical techniques, and regulatory needs converge to determine the quality and comparability of PSD data.

### Day 1 — dynamic light scattering

The first day established the conceptual and regulatory foundation through DLS-focused sessions. The day opened with Dr. Anna Schwendeman from the University of Michigan, Co-Director of the CRCG, who welcomed participants and introduced the CRCG’s mission to advance regulatory science and foster collaboration among FDA, academia, and industry. She explained that the FDA funded center was established in 2020 through a cooperative agreement between the University of Maryland and the University of Michigan to bridge knowledge gaps in the development and assessment of complex generic drug products. Dr. Schwendeman highlighted that particle-size analysis represents a critical and cross-cutting scientific challenge across multiple product categories where shared understanding among stakeholders can accelerate progress toward generic drug access. She encouraged attendees to engage actively across disciplines and use the workshop as an opportunity to translate technical insight into community practice.

**Fig. 1:**
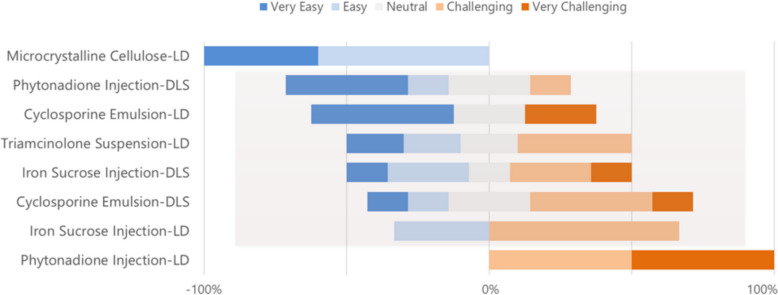
Survey results from the pre-workshop survey completed by participating vendors who performed pre-workshop sample measurement to assess their experience with evaluating each material: seven vendors participated, two techniques (DLS, LD), five materials, nineteen survey responses

Following the CRCG introduction, Dr. Xiaoming Xu from the FDA framed the workshop as a collaborative exploration of how particle-size data connect measurement principles with regulatory and industrial application. He emphasized that PSD measurement underpins product quality but is often misunderstood when treated as a single number rather than a context-dependent physical property. Dr. Xu reminded participants that measurement without purpose risks misinterpretation, and that robust results require clarity of intent, control of sample conditions, and transparency in reporting—a theme that would echo throughout the two-day program. Figure 1 illustrates results from the pre-workshop survey completed by participating vendors to assess their experience with evaluating the five pre-workshop materials. The findings revealed striking variability in how vendors rated the difficulty of measuring the same materials by DLS and LD. There was broad agreement that measurements with certain materials—such as microcrystalline cellulose by LD, which was largely viewed as easy, and phytonadione by LD, which was considered challenging —whereas the intermediate case studies revealed a more complex reality. Measurements such as phytonadione by DLS, cyclosporine by both LD and DLS, triamcinolone by LD, and iron sucrose by both techniques demonstrated a broad range of difficulty with PSD measurement even among these specialists in particle-size analysis. Dr. Xu noted that this diversity of expert opinion underscores the deceptively simple nature of particle-size measurement: what seems straightforward often conceals deep methodological differences shaped by formulation, technique, and interpretation.

Building on this conceptual foundation, Dr. Jeffrey Clogston from Nanotechnology Characterization Laboratory delivered the first plenary lecture, reviewing the physical principles that govern DLS and its application to nanoparticle and colloidal drug systems. He explained how temporal fluctuations in scattered-light intensity are translated into diffusion coefficients and hydrodynamic diameters and discussed how solvent viscosity, refractive index, and particle geometry affect these calculations (Bhattacharjee 2016; ASTM-E3247-20 2020; Hackley et al. 2010; Farkas and Kramar 2021). Through practical examples from product analysis, Dr. Clogston demonstrated that DLS measures ensemble behavior rather than individual particles, and that accurate interpretation requires awareness of the assumptions underlying the Stokes–Einstein relation. He emphasized the importance of experimental controls such as temperature stability, dilution, and scattering geometry to ensure reproducibility, reminding participants that the reliability of a DLS result depends as much on sample preparation as on instrument configuration.

Transitioning from measurement principles to regulatory examples, Dr. William Smith from the FDA discussed the FDA’s experience evaluating particle-size data submitted in complex generic applications. His presentation highlighted that analytical procedure validation for PSD typically encompasses repeatability, reproducibility and robustness, supported by explicit documentation of sample handling, instrument settings, and data-processing parameters. Variability in reported mean size, polydispersity index, or viscosity input values often complicates regulatory assessment, underscoring the need for clear communication of analytical assumptions. Dr. Smith emphasized that PSD characterization should be integrated within a broader product control strategy rather than used as a standalone specification, a perspective that aligns regulatory needs with scientific best practice.

The day continued with rapid technical presentations from seven instrument vendors (Bettersize Instruments, HORIBA, InProcess-LSP, Malvern Panalytical, Microtrac, part of Verder, Unchained Labs, and Waters/Wyatt Technology) each providing concise overviews of their DLS platforms and approaches to handling multimodal distributions. Anton Paar participated in the pre-workshop material analysis but did not present during the workshop. Data from the pre-workshop analysis are provided in Appendix A. Vendors described key instrument configurations, scattering-angle selections, and algorithms used to interpret complex samples. Shared challenges included managing multiple scattering, selecting appropriate dilution levels, correcting for viscosity, and interpreting intensity- versus volume-weighted data. Several vendors presented case examples using the pre-workshop materials, demonstrating how seemingly minor differences in sample handling or optical input parameters could lead to measurable variation in reported particle size. Collectively, these presentations illustrated that while hardware design contributes to precision, the primary drivers of variability remain methodological, reinforcing the need for transparent documentation and fit-for-purpose protocols.

**Fig. 2:**
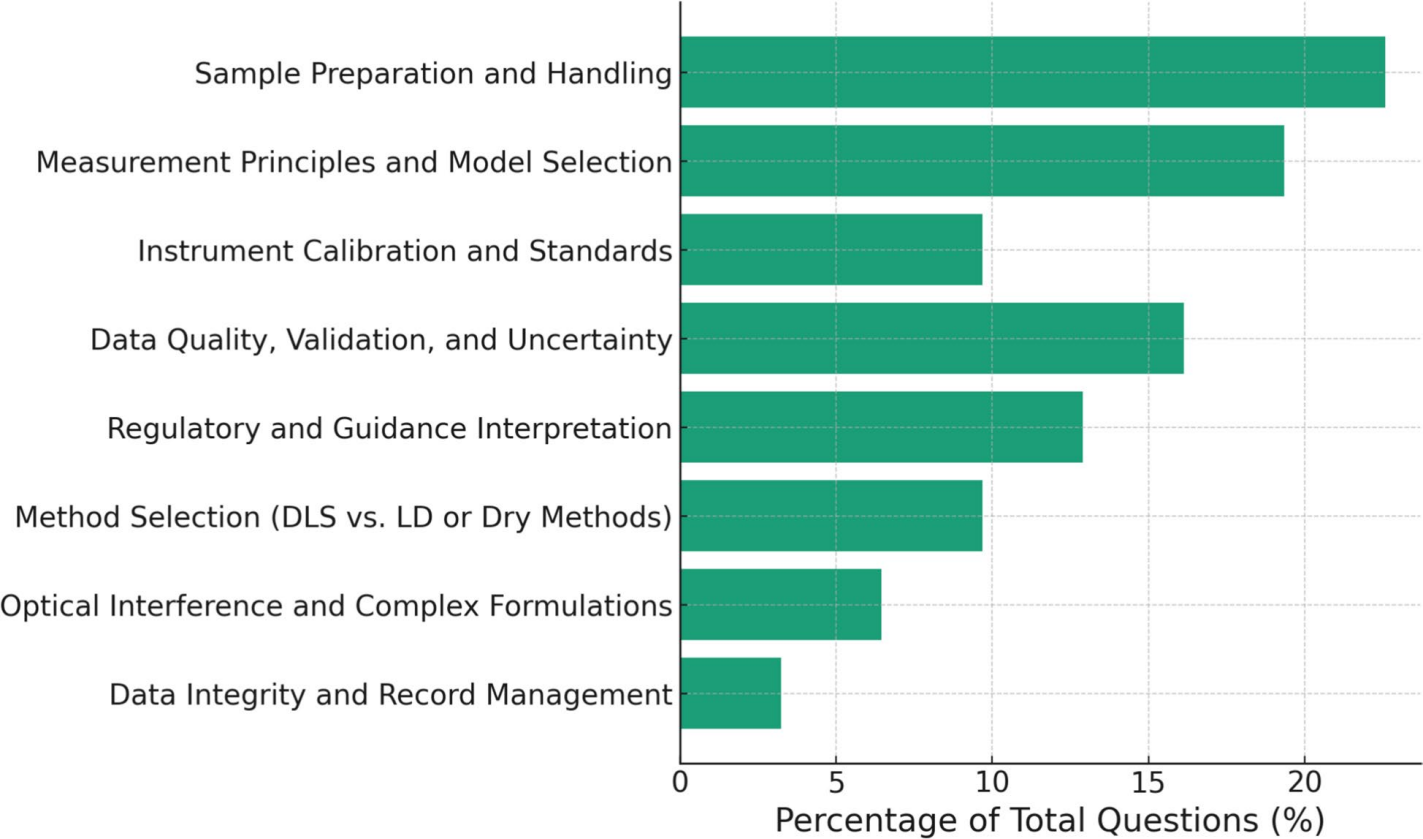
Distribution of online attendee questions that have been categorized based on content during the DLS panel discussion. Percentages were based on the total number of online questions for this session (*n* = 31, see Supplementary Materials Table S2 for more details)

Following these talks, a moderated panel discussion engaged speakers, vendors and participants in examining common sources of variability and strategies for improving robustness (see Table 1 and Fig. 2). The discussion addressed practical issues such as reconciling differences across instruments, confirming DLS data with orthogonal techniques like microscopy or nanoparticle-tracking analysis (NTA), and distinguishing instrumental precision from analytical relevance (Simon et al. 2023). Participants agreed that over-dispersion and excessive dilution can distort the true state of a formulation, highlighting the delicate balance between optical optimization and sample integrity. Sample preparation and handling questions were the most abundant questions asked from the online attendees (Fig. 2) The dialogue reinforced a central workshop message: consistent data interpretation depends on stronger communication between scientists, instrument vendors, and regulators.

**Table 1:** Summary of Panel Discussion – Day 1 Q&A (see Supplementary Materials Table S1 for more details)

DLS Topics	Core Insights
Viscosity Effects	• Inaccurate viscosity directly affects hydrodynamic size calculation • Measure under same experimental conditions • Avoid using default instrument values, instead measure dispersant viscosity for atypical dispersants such as surfactant-rich continuous phases, protein-containing formulations, or concentrated sugar-based vehicles, where viscosity deviates from water • Measure sample under multiple dilutions conditions
Weighting Modes	• Intensity vs. volume-weighted give different results • Choose based on analytical goal • Clearly report weighting type used
Instrument Variability	• Inter-vendor variability (light source wavelength, detection angle, and the optical model used) • Most variation comes from sample handling • Document preparation steps carefully • Distinguish real effects vs. procedural artifacts
Orthogonal Methods	• Microscopy or nanoparticle tracking analysis (NTA) can confirm DLS results • Use where appropriate for multimodal samples
Repeatability	• Context-dependent expectations • Focus on robustness, not identical values
Method Transfer	• Standardize preparation, mixing, temperature • Use internal reference materials for consistency
Regulatory Guidance	• Product-specific guidances (PSGs) should evolve with data • Emphasize collaboration and data sharing

The afternoon transitioned into a hands-on demonstration session where in-person attendees rotated among instrument stations to observe and discuss real-time DLS measurements on the pre-workshop materials. Participants interacted directly with vendor scientists to explore instrument setup, sample preparation steps, and data-acquisition parameters such as light source wavelength, detection angle, measurement duration, and dilution strategy. These live sessions provided an opportunity to compare how different systems approached the same formulation challenges—such as controlling multiple scattering in turbid samples or balancing dilution against signal intensity—and to see how parameter adjustments influenced resulting size distributions. The direct engagement allowed participants to appreciate both the technical capabilities of modern DLS instruments, and the nuanced decisions required for reliable measurements. Many attendees noted that seeing the measurement workflow in practice clarified sources of variability discussed earlier in the plenary sessions and reinforced the value of documenting each procedural step. Together, the demonstrations transformed conceptual learning into tangible experience, strengthening participants’ understanding of how instrument design, operator judgment, and sample behavior collectively shape PSD results.

Day 1 concluded with Session 4, small working group session in which in-person attendees gathered in small groups that included representatives from FDA, industry, vendors and academia to address predefined questions focusing on measurement purpose, method transfer, and data analysis (see Appendix B). This transition from lectures to collaborative discussion encouraged participants to apply the day’s conceptual lessons to practical case studies, setting the stage for identifying best practices in measurement robustness and comparability across laboratories. Expanded details and takeaways can be found in Sect. 3.

### Day 2 — laser diffraction

The second day opened with introductory remarks from Dr. Bin Qin from the FDA, who summarized key insights from the Day 1 discussions and introduced LD as a complementary tool for larger particle systems. He emphasized that both DLS and LD share fundamental challenges (dependence on optical models, refractive-index accuracy, and control of sample heterogeneity) and urged participants to adopt a “fit-for-purpose” mindset when designing or interpreting LD methods.

The morning plenary lecture by Dr. Alan Rawle, formerly with Malvern Panalytical, reviewed the theoretical and practical foundations of LD. He described how Mie scattering theory converts angular light-intensity patterns into volume-based particle-size distributions and demonstrated, through examples, how inappropriate refractive-index values, obscuration levels, or dispersion conditions can generate misleading artifacts (Beekman et al. 2005; Kippax 2005; Virden 2010; Keck and Muller 2008). Echoing his familiar observation that “*in theory, theory and practice agree; in practice, they do not*,”. Dr. Rawle stressed the necessity of experimental verification and cross-method validation to ensure that theoretical models align with empirical reality. As with all forms of microscopy, modern particle size instruments often require relatively low quantities of sample. However, achieving a statistically valid sample size with 1% standard error on the mean, requires 10,000 randomly selected particle images, emphasizing the importance of representative sampling from the bulk (Pitard 1989a; Pitard 1989b; ISO 14488:2007 2007; ISO 14488:2007/Amd 1:2019 2022; ASTM-E3440–22 2022). As a general rule, the minimum sample size for a 1% Fundamental Sampling Error (FSE) at a given x_99_ point in the distribution is illustrated in the table below (Rawle and Panalytical 2019) (Table 2):

**Table 2:** Minimum masses for 1% standard error at various x_99_ points in a particle size distribution

x_99_ (µm)	Approximate Minimum Mass *
1	-
10	1 mg
100	1 g
1000	1 kg
10,000	1000 kg

^*^Based on particle density 2.5 g/cm^3^

The program then transitioned to a series of industry case studies illustrating the application of LD to complex pharmaceutical formulations. Dr. Jernej Grmaš from Sandoz discussed challenges in determining active pharmaceutical ingredient (API) particle-size distribution within sub-micron gel suspensions intended for in vitro bioequivalence studies. LD analysis consistently showed a bimodal distribution confirmed by microscopy, with the smaller mode corresponding to the API and the larger to excipient gel structures. To isolate the API signal, Dr. Grmaš evaluated two complementary strategies: (1) using the placebo matrix for background subtraction to remove excipient interference (Vo et al. 2021) and (2) post-processing raw LD data to exclude the excipient peak. Each approach improved selectivity but introduced trade-offs—placebo subtraction requires exact formulation matching, whereas post-processing can compromise data integrity. His presentation underscored that excipient interference remains a central barrier to robust PSD method development and called for standardized guidance on handling multi-component suspensions.

Dr. Yousif Ayoub from Teva Pharmaceuticals examined the gap between laboratory practice and regulatory expectations, emphasizing the absence of clear guidance for LD method validation and specification setting. Drawing on patterns revealed through an AI-assisted review of FDA correspondence, he highlighted recurring agency requests for microscopy-based accuracy verification and variability in validation strategies across global development sites. The analysis prompted the creation of a working group to align internal best practices and promote consistency in documentation and justification. His presentation demonstrated how AI tools can identify systemic regulatory gaps and accelerate harmonization of PSD methodologies across organizations.

Mr. Rama Subba Reddy from Dr. Reddy’s Laboratories addressed challenges in DLS method development for heterogeneous formulations such as nano-emulsion creams and protein-containing dispersions. He showed that complex matrices often contain overlapping droplet and lamellar structures, producing multimodal distributions that are difficult to interpret with standard models. Case examples involving carrier proteins like human serum albumin (HSA) illustrated how self-association and bio-corona formation distort apparent particle size. He emphasized that reproducibility depends on careful control of concentration, dispersant, and attenuation settings and on integrating compositional knowledge with optical understanding.

Providing an industry-wide perspective, Mr. Bernard Domnic from Teva Pharmaceuticals discussed common regulatory challenges encountered in ANDA submissions involving particle-size data. He noted that inconsistencies in software algorithms, terminology, and validation formats frequently create miscommunication between applicants and assessors at regulatory agencies. Mr. Domnic advocated early engagement with regulators and transparent analytical justification to improve review efficiency and foster harmonized expectations for robustness testing and reporting. Together, these case studies reinforced that successful application of LD and DLS to complex drug products requires not only technical optimization but also shared understanding among formulation scientists, instrument developers, and regulators, a theme that echoed throughout the workshop.

The day continued with rapid technical presentations from four instrument vendors (Bettersize Instruments, HORIBA, Malvern Panalytical, and Microtrac) each providing concise overviews of their LD platforms and distinctive optical or analytical capabilities. The presentations compared approaches for controlling obscuration, selecting refractive-index inputs, and mitigating multiple-scattering effects in concentrated or highly absorbing samples. Several vendors demonstrated how dispersion control, stir speed, and circulation geometry influence reproducibility, and highlighted features such as automated alignment and live feedback that help maintain stable optical conditions. Collectively, these rapid-fire talks illustrated that while hardware configurations differ, the dominant sources of measurement variability remain procedural, emphasizing again that consistent sample handling and parameter documentation are key to comparability across instruments and laboratories.

**Fig. 3:**
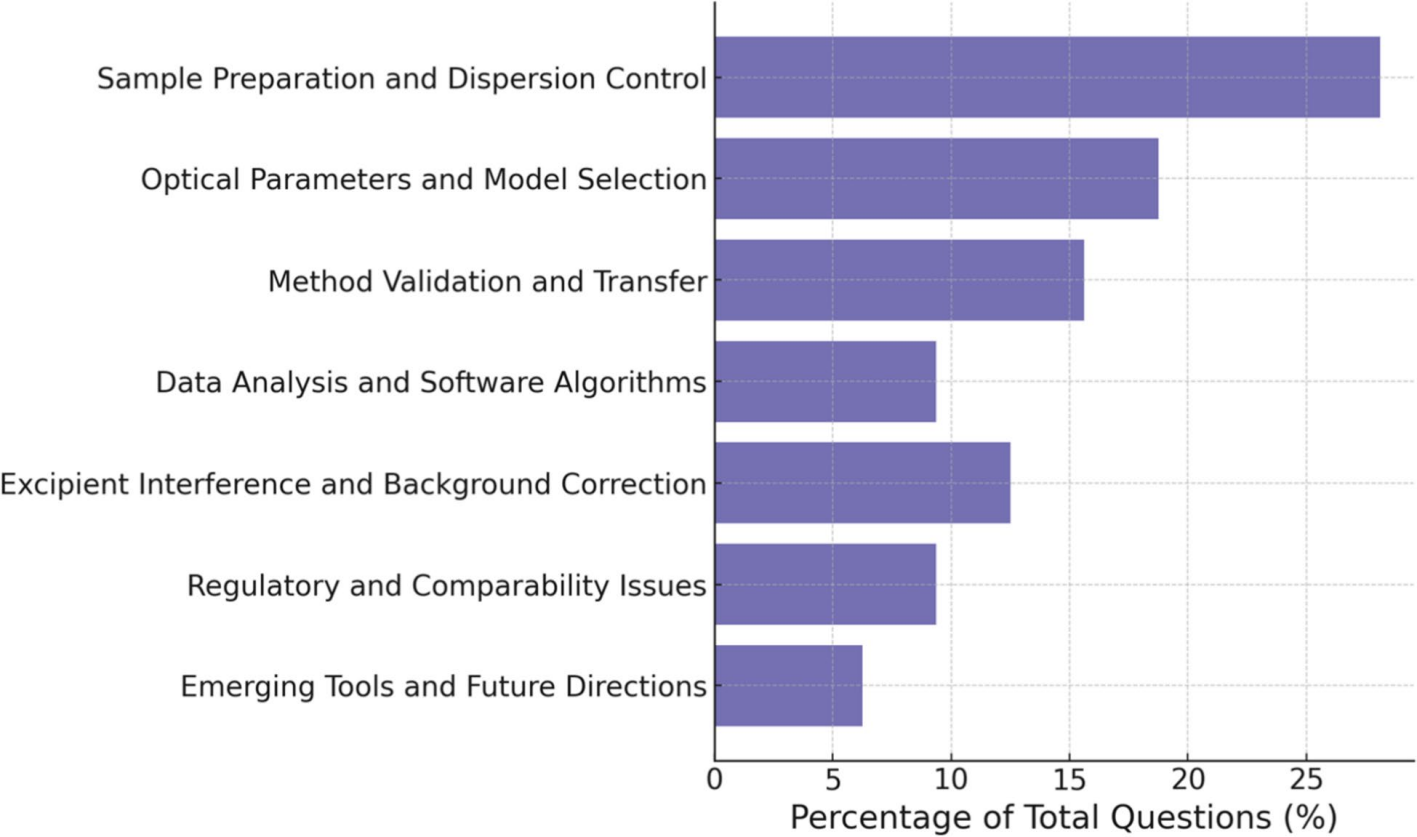
Distribution of online attendee questions categorized based on content during the LD panel discussion. Percentages were based on the total number of online questions for this session (*n* = 32, see Supplementary Table S4 for details)

A moderated panel discussion followed, featuring vendor representatives together with industry and regulatory participants (Table 3 and Fig. 3). The dialogue explored challenges in achieving inter-platform alignment, particularly when proprietary algorithms and reporting formats differ. Panelists agreed that transparent disclosure of processing parameters is more valuable than uniformity of instrument design, and that collaboration through shared datasets and inter-laboratory comparisons would advance harmonization. Questions from the audience focused on handling multimodal distributions, selecting between wet and dry dispersion modes, and managing highly viscous or surfactant-rich formulations. The discussion concluded with consensus that progress in particle-size analysis depends on open communication between developers, users, and reviewers, echoing the workshop’s broader theme of “fit-for-purpose” measurement and shared scientific understanding.

**Table 3:** Summary of panel discussion – Day 2 Q&A (see Supplementary Table S3 for details)

LD Topics	Core Insights
Excipient Interference	• Transparency and justifications are key • Evaluate placebo-background subtraction methods • Post-workshop measurements planned
Refractive Index Issues	• Estimate based on literature or component weighting • Perform sensitivity analysis to show robustness
Dispersion Stability	• Control stir speed and surfactant level • Avoid foaming and air bubbles • Document dispersion conditions
Software Transparency	• Disclose smoothing/deconvolution settings • Focus on transparency over strict standardization
Comparability & Regulation	• Use RLD lots when data-limited • Ensure clear rationale for comparability • Purpose is reproducibility, not to enforce rigid number

Plenary closing remarks wrapped the conference for virtual attendees, integrating lessons learned from both DLS and LD. Participants agreed that the two techniques provide complementary insights across distinct particle-size regimes and that accurate interpretation requires contextualizing each measurement within its analytical purpose, optical assumptions, and formulation properties. Recurrent themes included the need for standardized reporting templates, improved cross-sector communication, and sustained collaboration among instrument developers, users, and regulators.

The afternoon featured live LD demonstrations by vendor representatives using the same materials analyzed by DLS on Day 1 for in-person attendees. Participants observed firsthand how sample foaming, over-obscuration in concentrated suspensions, and inaccurate refractive-index inputs affect calculated distributions. Comparing real-time data processing across systems helped attendees appreciate how instrument configuration, software algorithms, and operator choices jointly influence results. The demonstrations reaffirmed that reproducibility depends as much on transparent parameter reporting and careful sample handling as on instrument design itself.

Day 2 talks and live-demonstrations provided a conceptual bridge to pre-workshop data-specific small working group discussions in Session 9 with in-person attendees and the final Waypoint Exercise, which synthesized the workshop’s collective findings into priorities for future work. Expanded details and takeaways from these exercises can be found in Sects. 4 and 5.

## Small working group session on measurement and data interpretation

Following the plenary lectures, participants engaged in structured small working group exercises designed to translate conceptual understanding into practical insight during Session 4. Tables were organized to include scientists from regulatory, academic, industrial, and vendor backgrounds, ensuring diverse perspectives in each discussion. Using questions drawn directly from the session handouts (see Appendix B), the groups examined how measurement purpose, method robustness, materials and standards, data comparison, and communication practices collectively influence the reliability of PSD results.

### Clarifying the purpose of measurement

Participants emphasized that meaningful PSD data collection must begin with a clearly articulated analytical purpose (e.g., using an analytical target profile ICH Q14), since the goal of measurement determines every subsequent methodological choice. Measurements aimed at formulation development or mechanistic understanding demand high sensitivity and resolution, whereas those intended for routine quality control prioritize simplicity, repeatability, and speed. Participants noted that this purpose is often implicitly shaped by product context, including dosage form and route of administration, which in turn influences which particle-size attributes are most relevant for interpretation. Without a defined objective, results risk losing interpretive value: excessive dilution or over-dispersion may stabilize optical signals but change native particle structures, while insufficient mixing can obscure smaller populations. Every preparatory parameter—dispersant selection, sonication energy, mixing rate, equilibration time—should therefore be justified against the measurement’s purpose and recorded transparently. A well-stated purpose transforms numerical outputs into evidence capable of supporting process understanding, product performance, or regulatory comparison.

### Ensuring robustness, transferability, and validation

Robustness and transferability emerged as recurring priorities. Participants defined robustness as the ability of a method to produce consistent results under small, deliberate variations in sample preparation or instrument setting, and transferability as reproducibility of those results across laboratories and/or across instrument models. These qualities, they noted, must be demonstrated empirically rather than assumed from manufacturer specifications. Inter-laboratory failures were often traced to “hidden variables,” such as vial geometry, pre-mix time, or temperature control, that were undocumented yet influential. Validation was described as the systematic accumulation of evidence—repeatability, intermediate precision, sensitivity testing—showing the method performs as intended across its range. Participants recommended detailed documentation of experimental parameters and use of internal reference dispersions to maintain consistency among sites. Overall, robustness and validation were viewed not as procedural requirements but as essential pillars of credible measurement science.

### Materials and standards for method development

Discussion of materials highlighted a persistent need for reference dispersions that better represent pharmaceutical systems. Existing bead or latex standards are useful for instrument calibration but do not capture the optical or rheological complexity of real formulations. Participants identified this gap as a major barrier to method comparability across laboratories. Several tables proposed developing well-characterized “fit-for-purpose” reference emulsions or suspensions through collaborative CRCG or academic partnerships. These materials should have traceable viscosity, refractive-index, and polydispersity values, enabling standardized benchmarking of both DLS and LD methods. Establishing shared datasets linking such standards to validated protocols was viewed as a practical step toward inter-laboratory alignment.

### Evolving methodologies for future needs

Looking ahead, participants discussed how particle-size methodologies must evolve to support emerging manufacturing paradigms and regulatory needs. Process-analytical technologies capable of in-process or at-line monitoring will become integral to continuous manufacturing, demanding PSD methods that can operate under dynamic conditions while preserving data traceability. Participants foresee increasing reliance on automation, real-time feedback, and integrated metadata capture to strengthen data integrity. They also emphasized that transparent software algorithms and advanced data-analytics tools will be essential for interpreting complex or multimodal distributions. Sustained progress will depend on interdisciplinary expertise that bridges formulation science, optics, physics of molecular scattering, and statistics, transforming particle-size analysis from a laboratory assay into a cornerstone of process understanding.

### Comparing results across batches and laboratories

When comparing PSD data, participants agreed that full-distribution analysis provides greater insight than reliance on single-point metrics such as D10, D50, or D90. Overlaying cumulative or differential curves allows visualization of distribution shape, modality, and tails, revealing subtle differences that averages may conceal. Quantitative metrics such as earth-mover distance (EMD) can supplement visual inspection, but graphical comparison and expert judgment remain indispensable. More broadly, participants noted that orthogonal analytical approaches—particularly low-throughput or imaging-based methods—can provide valuable contextual information when interpreting complex or heterogeneous particle-size data (Wilson and Prud'homme 2021). A tolerance of ± 10% for key percentiles was considered reasonable for inter-laboratory comparisons when sample preparation and optical conditions are equivalent. The consensus was that meaningful comparison integrates statistical and visual assessment to ensure numerical similarity reflects genuine physical equivalence.

### Communicating uncertainty and limitations

Transparent communication of variability was recognized as central to scientific integrity. Participants recommended that PSD results always report replicate statistics, means with standard deviations or relative standard deviations, along with details of replicate count, instrument model, and operator. Graphical displays, such as shaded error bands, were encouraged to illustrate data spread clearly. Distinguishing between measurement uncertainty (instrumental or procedural) and sample heterogeneity (material-intrinsic) provides critical context for data interpretation and regulatory review. Embracing uncertainty as an inherent property of measurement to be quantified, rather than a deficiency to minimize, was viewed as key to promoting reproducibility and confidence in reported data.

### Improving collaboration and communication

Participants unanimously emphasized that stronger collaboration among industry, vendors, academia, and regulators is essential to achieving consistency in PSD measurement. Many discrepancies arise from differences in terminology, reporting format, or interpretive assumptions rather than equipment capability. Practical mechanisms to close these gaps included collaborative ring studies using shared samples, joint training workshops, and open data repositories to reveal systematic biases. Instrument manufacturers were encouraged to increase transparency regarding algorithm details and participate actively in community benchmarking exercises. Such cooperative learning environments were seen as vital for building mutual understanding and accelerating harmonization.

### Toward harmonized expectations

The discussions concluded with reflection on what harmonized expectations should entail. Participants agreed that harmonization does not require identical instruments or procedures, but a shared framework defining analytical equivalence and transparent reporting. Immediate priorities included consensus terminology, standardized templates for reporting sample history and optical parameters, and clear disclosure of data-processing steps. The idea of a joint FDA–CRCG white paper received strong support as a means to codify these expectations and propose a roadmap for continued collaboration. Participants recognized that true harmonization would arise gradually through transparency, shared vocabulary, and coordinated studies that align scientific understanding across the measurement community.

## Small working group session on sample-specific discussions

Session 9 of the workshop applied the concepts and lessons developed during earlier sessions to the interpretation of real data from three pre-workshop materials: cyclosporine emulsion, iron sucrose colloidal dispersion, and triamcinolone suspension. Each group analyzed pre-workshop vendor measurements, discussed observed variability, and proposed explanations rooted in formulation characteristics and optical theory. The aim was to connect measurement outcomes to scientific reasoning and to identify common principles for improving reproducibility. Only three of the five pre-workshop materials were included in these discussions because they represented distinct formulation types—emulsion, colloidal dispersion, and suspension—offering the most instructive contrasts in measurement challenges and data interpretation. The remaining two samples were not covered due to time limitations and their less differentiated measurement behavior across techniques.

### Cyclosporine emulsion

Discussions around the cyclosporine emulsion centered on understanding why laboratories obtained divergent particle-size distributions despite using comparable DLS and LD instruments. Participants noted that DLS data commonly exhibited two distinct populations, a narrow peak near 20 nm and a broader distribution between 100 and 150 nm, corresponding respectively to micellar surfactant assemblies and dispersed oil droplets. LD results showed wider or sometimes multimodal profiles, reflecting sensitivity to turbidity, viscosity, and foaming. Apparent size inflation was frequently traced to multiple scattering and bubble entrapment, both of which distort optical paths and mislead automated algorithms. Groups emphasized that controlled dilution, typically 50–100 fold, improved reproducibility by balancing scattering intensity without disrupting emulsion integrity. Accurate viscosity measurement was identified as critical, since DLS calculations convert diffusion coefficients to hydrodynamic diameters via the Stokes–Einstein relation; neglecting the true viscosity of surfactant-rich media can bias results by tens of nanometers. Best-practice recommendations included using backscatter detection to mitigate multiple-scattering errors, employing low-shear mixing to minimize foam, and verifying stability through repeat measurements over time. Participants concluded that cyclosporine emulsions exemplify the broader lesson that optical turbidity and compositional complexity magnify procedural differences, making transparent reporting of every preparation step essential for cross-laboratory comparability.

### Iron sucrose colloidal dispersion

For the iron-sucrose colloidal dispersion, participants confronted the analytical difficulties posed by a highly absorbing, viscous liquid whose optical and rheological properties depart from standard model assumptions. The sample’s dark coloration and dense sucrose matrix generated low scattering intensity and unstable baselines in DLS, while LD instruments suffered from light attenuation and stray-light artifacts. Reported particle sizes ranged from 10 to 40 nm, with inconsistent repeatability even under nominally identical conditions. Group analysis linked these discrepancies to wavelength-dependent absorption of the ferric complex, to localized photothermal effects from high-power or short-wavelength lasers that can induce aggregation, and contributions in DLS signal from nanoparticles that are contaminants native to the sucrose (Weinbuch et al. 2015; Kaszuba et al. 2007). Because elevated viscosity alters Brownian motion, size measurement values are temperature-sensitive. Recommended mitigation strategies included modest dilution (× 10–50 in isotonic saline) to reduce viscosity without destabilizing the colloid, coupled with longer-wavelength or lower-intensity illumination to minimize sample heating from incident light. Participants emphasized explicit reporting of measurement wavelength, dilution factor, and temperature to enable meaningful comparison. Because day-to-day reproducibility proved more informative than single-run precision, averaging multiple independent runs was proposed as a realistic approach for such absorbing systems. Overall, the iron-sucrose discussions highlighted the limits of one-size-fits-all protocols and reinforced the need for contextual interpretation and transparency when analyzing optically complex colloidal dispersions.

### Triamcinolone suspension

The triamcinolone suspension discussions focused on the dual challenge of achieving uniform dispersion of micronized solids while preserving their native particle structure. LD measurements typically showed a primary mode near 6–8 µm with a minor coarse tail, whereas microscopy measurements showed that apparent bimodality often resulted from incomplete de-agglomeration rather than distinct particle populations. Participants observed that sonication energy, dispersant composition, and mixing time exerted the greatest influence on reproducibility: sonication fragmented soft agglomerates and reduced mean size, while insufficient dispersion led to particle clusters that inflated D90 values. Variation in results across laboratories frequently stemmed from dissimilarity in dispersion protocols: differences in surfactant concentration, solvent composition, or shear rate. To mitigate these effects, groups recommended specifying both the duration and amplitude of sonication (for example, 60 s at a fixed setting). Selecting an appropriate dispersant was also critical: aqueous surfactant media improved wetting for hydrophobic powders but could dissolve surface stabilizers and alter optical contrast, shifting the refractive-index assumptions in LD models. Participants agreed that suspension analysis demands a holistic protocol integrating sample handling, optical parameters, and verification steps. Standardized preparation and reporting—including documentation of energy input, medium composition, and pre-measurement observations—were viewed as essential for improving inter-laboratory comparability and ensuring that PSD results accurately represent formulation behavior.

## Waypoint exercise — collective reflection and future directions

The workshop concluded with a structured “Waypoint” reflection in which participants, grouped by table, summarized the five most important lessons or recommendations they derived from two days of discussion and hands-on analysis. This exercise transformed individual experiences into collective insight, highlighting convergent priorities across sectors and disciplines. Despite participants’ varied backgrounds—regulatory, industrial, academic, and vendor—the reflections showed striking consistency, revealing shared recognition of the technical and organizational challenges that shape particle-size measurement today.

A dominant theme across nearly all tables was the need for *standardization and harmonization in both terminology and reporting*. Participants agreed that variability across laboratories often arises less from instrument performance than from differences in assumptions, optical inputs, and documentation. They called for a unified reporting template capturing key contextual details such as sample history, refractive-index and viscosity values, optical model parameters, and data-processing algorithms. Establishing such a framework would promote transparency and enable meaningful cross-study comparison. Several groups specifically proposed that FDA and CRCG coordinate development of a community white paper outlining minimal expectations for PSD method description, validation, and reporting—an initiative viewed as a practical step toward lasting harmonization. There have also been international efforts to modernize analytical procedure development and validation protocols with specific examples provided in ICH Q2(R2) training materials for particle size measurements as a property test for pharmaceuticals (ICH Q2(R2) Training Materials Module 3). The concept of an inter-laboratory study using shared reference materials also gained wide support as a mechanism to quantify variability and benchmark comparability among instruments and methods.

A second recurrent theme concerned *the importance of communication and collaboration across stakeholder communities*. Participants observed that many analytical discrepancies arise not from equipment limitations but from differences in interpretation, training, or even vocabulary. They emphasized that open dialogue between instrument developers, users, and regulators is essential for improving understanding of both measurement principles and regulatory expectations. Suggestions included recurring joint workshops, technical webinars, and open-access data repositories where anonymized datasets could be shared for educational and benchmarking purposes. Such sustained engagement was viewed as the most effective means of building mutual trust and accelerating convergence of analytical practice across the pharmaceutical landscape.

A third emphasis was placed on *education, training, and knowledge transfer*. Attendees praised the workshop’s interactive design — combining lectures, demonstrations, and collaborative working-group sessions — and recommended institutionalizing similar formats through future CRCG initiatives. They noted that particle-size measurement sits at the intersection of formulation science, optics, and statistics, and that interdisciplinary expertise is essential for interpreting complex data responsibly. Structured training programs, laboratory exchanges, and case-based learning modules were proposed to strengthen technical proficiency and ensure continuity of best practices across generations of scientists. Participants recognized that the next stage of progress will depend on developing a workforce capable of bridging theory, instrumentation, and regulatory application.

Another recurring reflection highlighted *data integrity and transparency as cornerstones of reliable measurement*. Participants underscored the growing importance of digital audit trails, metadata capture, and version-controlled analysis workflows in ensuring reproducibility and regulatory confidence. Integrating these capabilities into future instrument software and data-management platforms was seen as a logical evolution of good measurement practice. This emphasis on integrity extended beyond technology to scientific culture—reinforcing that transparency, not perfection, is the true hallmark of credible data.

Together, these reflections painted a clear trajectory for the field: particle-size analysis must evolve from a collection of techniques into a cohesive, community-driven discipline grounded in shared standards and continuous learning. Participants viewed the workshop not as a conclusion but as the beginning of a collaborative effort to strengthen the scientific and regulatory foundations of PSD measurement. The collective enthusiasm expressed during the Waypoint session demonstrated the community’s readiness to continue this work through follow-up publications, inter-laboratory studies, and future FDA–CRCG partnerships dedicated to advancing measurement science.

## Conclusions

The Mastering Particle Size Analysis workshop demonstrated that the greatest challenges in PSD measurement arise not from instrumental limitations but from differences in intent, sample handling, and data interpretation. Across all sessions, participants converged on a shared understanding that reliable particle-size data depend on defining the measurement’s purpose, maintaining transparent documentation, and contextualizing numerical results within the physical and formulation realities of the system under study. The workshop reaffirmed that DLS and LD are not competing methods but complementary tools that, when applied judiciously, can together provide a more complete understanding of product microstructure.

Through presentations, vendor demonstrations, and interactive working group sessions, the workshop identified the core elements that determine data reliability—fit-for-purpose method design, validation through controlled variation, and clear communication of uncertainty. The small working group exercises and sample-specific analyses highlighted how variations in optical parameters, viscosity assumptions, and dispersion protocols can lead to divergent results even when nominally identical materials are tested. These findings reinforced the necessity of harmonized terminology, transparent reporting, and collaborative verification to ensure that PSD data are interpretable and reproducible across laboratories.

The concluding Waypoint reflection provided a unifying vision for the future: that progress in particle-size analysis will depend as much on communication and collaboration as on instrumentation. Participants articulated a collective commitment to advancing harmonization, promoting open data exchange, and developing shared training and reference materials through coordinated FDA–CRCG efforts. The strong alignment across regulators, industry scientists, academics, and instrument developers signaled a maturing consensus that particle-size analysis is not merely a technical exercise but a discipline of interpretation where the meaning of a number depends on understanding the system, the model, and the purpose behind every measurement.

In this sense, the workshop achieved more than the transfer of knowledge, it fostered a renewed scientific culture of transparency, reflection, and cooperation. The outcomes underscore that meaningful particle-size data emerge when measurement, theory, and context are united. By transforming dialogue into action, the community is now positioned to continue this collaboration through future studies, publications, and educational initiatives that will shape the next generation of best practices in pharmaceutical particle-size characterization.

## Supplementary Information

Supplementary Material 1.

## Data Availability

All presentations and conference associated materials can be found on CRCG website: https://www.complexgenerics.org/education-training/mastering-particle-size-analysis-a-step-by-step-illustration-of-techniques-and-best-practices/.

## Appendix A

### Vendor reports on pre-workshop sample measurements

FDA‐CRCG Public Workshop, Vendor Data/Reports Tab: Mastering Particle Size Analysis: A Step-By-Step Illustration of Techniques and Best Practices, September 23, 2025. Accessed November 3, 2025. https://www.complexgenerics.org/education-training/mastering-particle-size-analysis-a-step-by-step-illustration-of-techniques-and-best-practices/.

## Appendix B

### Small Group Work Session Questions

#### Session 4 Theme 1: Measurement

- Question 1: How does defining the measurement purpose influence method selection, sample preparation, and data interpretation?
  - Differences in needs across product development, quality control, and bioequivalence studies.
  - Ties between measurement purpose and specification setting (e.g., NMT vs.±%).
  - When particle size acts as a proxy for product performance (e.g., dissolution, processing).
  - Barriers to method selection (e.g., unclear requirements, vendor communication gaps).
  - Avoiding “garbage in = garbage out” through early alignment of goals and methods. 
- Question 2: What factors determine whether a particle size method is robust, transferable, and validated?
  - Criteria for robustness in light scattering or laser diffraction techniques.
  - Managing cross-instrument or cross-technique variability—how much difference is acceptable?
  - Challenges in method transfer (e.g., lab-to-lab, CRO to sponsor, development to QC).
  - Method change: when is revalidation required, and to what extent (partial vs. full)?
  - Extent of permissible parameter adjustments during method transfer and their impact on comparability and robustness.
- Question 3: What materials and standards support method development, and where are the current gaps?
  - Choosing between real product, surrogate materials, or reference standards.
  - Limitations and variabilities in sourcing materials (e.g., RLD, ANDA product, or NIST-traceable).
  - Wishlist for ideal materials, and how these affect precision or bias.
  - What’s missing in current technology, and how can emerging tools fill the gap?
- Question 4: What future directions should measurement methodologies take to meet evolving needs?
  - Technological limitations of current platforms (e.g., resolution, sample prep complexity, algorithm dependence).
  - Ideas for next-generation technologies and methods?
  - Translating novel methods to routine QC—barriers and enablers.
  - Research needed to close measurement gaps or enable new applications.
  - Challenges in regulatory acceptance of novel techniques.

#### Session 4 Theme 2: Data Analysis, Comparison, and Reporting

- Question 1: What’s the most meaningful way to compare particle size results across batches or labs?
  - Limitations and uses of population bioequivalence, EMD, and other statistical approaches.
  - Deciding on number-, volume-, or intensity-based reporting for different settings.
  - Managing variation: what counts as “same enough” in different contexts?
  - QC vs. BE studies—do different use cases require different acceptance criteria?
  - What is the relative importance of each “accuracy” and “precision” when the true size distribution is unknown
  - Complementary vs Orthogonal? What is the importance of a complementary method? What is the importance of an orthogonal method?
- Question 2: How should uncertainty and limitations be communicated in regulatory or technical documents?
  - Strategies for reporting limitations transparently without affecting approvals.
  - Explaining analytical uncertainty and assumptions to reviewers.
  - Role of common terminology and formats in improving mutual understanding.
  - Examples of effective vs. problematic communication in past filings.
- Question 3: How can we improve collaboration and communication across stakeholders (industry, vendors, regulators)?
  - Identifying systemic barriers to open dialogue and knowledge sharing.
  - Opportunities for precompetitive collaboration or forums (e.g., working groups).
  - How shared terminology, visual tools, or reporting templates can bridge gaps.
  - Role of vendors in method development, validation support, and post-approval changes.
- Question 4: What would harmonized expectations for particle size methods look like—and how do we get there?
  - Existing standards (e.g., ISO, ASTM)—how helpful are they? Where do they fall short?
  - Adequacy of current FDA’s Product Specific Guidances —where is more clarity needed?
  - Global harmonization vs. regional differences—what’s realistic? What role can academic-industry-regulator partnerships play in setting norms?

## Abbreviations

3R: Repeatability, Reproducibility, and Robustness
AI: Artificial Intelligence
ANDA: Abbreviated New Drug Application
API: Active Pharmaceutical Ingredient
ASTM: American Society for Testing and Materials (referenced in standards)
BE: Bioequivalence
CRCG: Center for Research on Complex Generics
CRO: Contract Research Organization
D10, D50, D90: Particle size distribution percentiles (10th, 50th, and 90th percentiles)
DLS: Dynamic Light Scattering
EMD: Earth-Mover Distance
FDA: U.S. Food and Drug Administration
FSE: Fundamental Sampling Error
HSA: Human Serum Albumin
ICH: International Council for Harmonisation
ISO: International Organization for Standardization
LD: Laser Diffraction
MCC: Microcrystalline Cellulose
NIST: National Institute of Standards and Technology
NMT: Not More Than (specification type)
NTA: Nanoparticle-Tracking Analysis
PBE: Population Bioequivalence
PSD: Particle-Size Distribution
PSG: Product-Specific Guidance
QC: Quality Control
RI: Refractive Index
RLD: Reference Listed Drug
USG: The Universities at Shady Grove
USP: United States Pharmacopeia
