# Supplementary material for: Mastering particle size analysis: lessons, challenges, and future directions from the FDA–CRCG workshop
Source: AAPS Open. 2026 Apr 6;12(1):11. doi: 10.1186/s41120-026-00148-4 (PMC13098874; doi:10.1186/s41120-026-00148-4)
Supplement: Supplementary file 1 — Supplementary Material 1. [file 41120_2026_148_MOESM1_ESM.docx]

**Supplementary Materials**

**Mastering Particle Size Analysis: Lessons, Challenges, and Future Directions from the FDA–CRCG Workshop**

Xiaoming Xu^1^*, William C. Smith^1^, James E. Polli*^2^, Christine L. Plavchak^1^, Bin Qin^3^, Yan Wang^3^, Nicholas Holtgrewe^1^, Sam G. Raney^3^, Andre O’Reilly Beringhs^3^, Bruce Yu^4^, Marc Taraban^4^, Vishalakshi Krishnan^5^, Anna Schwendeman^5^, Dana C. Hammell^2^, Jeffrey D. Clogston^6^, Alan F. Rawle^7^, Jernej Grmaš^8^, Yousif Ayoub^9^, Bernard Domnic^10^, Rama Subba Reddy^11^, Joseph Duncan^12^, Matthew McGann^13^, Jeff Bodycomb^14^, Carl C.L. Schuurmans^15^, Michiel Hermes^15^, Jonathan G. Mehtala^16^, Prashun Roy^17^, Kevin Lance^18^, Collin Britten^19^, Kiwan Park^20^**,** Daniel Beach^16^, Matthew Elmer^17^**,** Keith B. Rodenhausen^21^, Zhen Xu^3^, Haiou Qu^1^, Kai-Wei Wu^3^, Maxwell Korang-Yeboah^1^, Yuan Zou^3^, Ji Li^5^, Alaa Abuznait^3^, Colette Quinn^22^, Anabelle Ethier^23^, Kendice Ip^24^, Dinesh Dhamecha^3^, Qi Li^3^, Dongkai Zhu^1^, Rutu Valapil^2^

*Corresponding authors: Xiaoming Xu ([xiaoming.xu@fda.hhs.gov](mailto:xiaoming.xu@fda.hhs.gov), 240-495-2094), James E. Polli ([jpolli@rx.umaryland.edu](mailto:jpolli@rx.umaryland.edu), 410-706-8292)

**Table S1.** Extended summary of panel discussion on DLS– Day 1 Q&A

| **Question** | **Summary of Panel Response** |
| --- | --- |
| 1. How do we interpret particle-size data when viscosity or zero-shear viscosity varies across samples, and should it always be measured directly rather than assumed? | Viscosity strongly influences calculated hydrodynamic diameters. It should be measured or estimated under the same experimental conditions rather than using instrument defaults. Accurate input values are critical for meaningful DLS results. |
| 2. How should analysts handle the choice between intensity-weighted and volume-weighted distributions when reporting DLS data? | The two weighting modes emphasize different physical aspects; results are not interchangeable. Analysts should state clearly which representation is used and ensure consistency with the analytical goal and technique. |
| 3. What is the acceptable range of variability between instruments or laboratories, and how can true material differences be distinguished from methodological artifacts? | Most variability arises from sample handling rather than instrument design. Detailed documentation of preparation steps—dilution, mixing, sonication, and equilibration—is essential to separate real sample effects from procedural differences. |
| 4. To what extent can orthogonal techniques such as microscopy or nanoparticle-tracking analysis serve as confirmatory tools for DLS? | Orthogonal methods provide valuable confirmation, especially for multimodal or highly polydisperse systems, but should be applied based on analytical purpose rather than mandated use. |
| 5. What level of repeatability or precision should be expected for complex emulsions or colloidal dispersions? | Expectations must be context-dependent. Complex or turbid systems inherently show greater variability; robustness—reproducible results under controlled variation—is more meaningful than identical values. |
| 6. How can method-transfer protocols minimize variability from uncontrolled factors such as mixing energy, sonication, or temperature? | Standardizing preparation and documenting environmental and mechanical parameters are key. Using internal reference materials or cross-checks helps ensure continuity across laboratories. |
| 7. Should product-specific guidances (PSGs) provide clearer recommendations on preferred techniques and required parameter standardization? | Guidance should evolve with new data. PSGs aim to balance clarity with flexibility, encouraging continued dialogue and data sharing to inform future updates. |

**Table S2.** Overview of Online Attendee Questions – Day 1 (Dynamic Light Scattering Focus)

| **Category** | **Representative Question Topics** | **Number of Questions** | **General Trend or Interest** |
| --- | --- | --- | --- |
| **1. Sample Preparation and Handling** | Filtration practices; dilution impact; effect of sonication or mixing; choice of dispersant; sedimentation vs. Brownian motion | **7** | Many attendees sought practical guidance on how sample preparation influences data accuracy and reproducibility. They expressed uncertainty about best practices for dilution, filtration, and maintaining dispersion stability. |
| **2. Measurement Principles and Model Selection** | Intensity- vs. volume-weighted results; reporting D10/D50/D90; optical model assumptions; handling non-spherical or rod-shaped particles | **6** | Participants wanted clarity on data interpretation and on how theoretical models translate to reportable metrics, particularly for irregular or multimodal systems. |
| **3. Instrument Calibration and Standards** | Use of size standards; instrument verification; USP chapters; frequency of calibration | **3** | Questions reflected interest in available standards (e.g., USP <429>) and acceptable approaches to verify instrument performance. |
| **4. Data Quality, Validation, and Uncertainty** | Accuracy tests; repeatability across instruments; robustness under varying conditions; handling out-of-spec data; acceptable variability | **5** | Many queries highlighted the challenge of quantifying measurement uncertainty and defining pass/fail criteria for validation. |
| **5. Regulatory and Guidance Interpretation** | Application of ICH Q2(R2); expectations for accuracy and orthogonal confirmation; interpretation of “span” or PBE tolerance; comparability across RLDs | **4** | Attendees sought to understand regulatory expectations and how to demonstrate equivalence or robustness within submissions. |
| **6. Method Selection (DLS vs. LD or Dry Methods)** | When to choose DLS or LD; appropriateness of dry measurement; combining techniques for primary vs. aggregate analysis | **3** | Participants wanted practical advice on selecting or combining PSD methods to characterize complex formulations effectively. |
| **7. Optical Interference and Complex Formulations** | Refractive-index mismatch; absorption (e.g., iron-sucrose); managing turbidity | **2** | Fewer but more advanced questions pointed to difficulties in analyzing highly absorbing or turbid samples. |
| **8. Data Integrity and Record Management** | Electronic data reliability; traceability; regulatory compliance | **1** | Participants recognized the importance of trustworthy digital records, echoing broader regulatory priorities. |

**Table S3.** Extended summary of panel discussion on LD – Day 2 Q&A

| Question | Summary of Panel Response |
| --- | --- |
| 1. How should excipient interference in LD data be addressed—through data truncation or placebo-background subtraction? | The panel did not take a position on the validity of either approach but emphasized the importance of transparency, justification, and documentation when applying any corrective method. Participants agreed to further review the published placebo-background subtraction method and assess its scientific basis. Following the workshop, all LD vendors were invited to conduct additional evaluations, with a post-workshop study planned using brinzolamide ophthalmic suspensions to explore this question in greater depth. |
| 2. When three manufacturing lots are unavailable, can an RLD serve as one lot to meet the “3 R” expectation for validation or comparability? | The goal of the three-lot principle is to demonstrate reproducibility, not to enforce a rigid number. Using an RLD lot can be appropriate when justified scientifically, provided analytical consistency and rationale are clearly explained. |
| 3. How can laboratories establish comparability across LD instruments when refractive-index data for excipients are uncertain? | Estimating refractive-index values based on literature or component weighting is acceptable if assumptions are documented. Sensitivity analysis showing that reasonable variations do not change conclusions strengthens credibility. |
| 4. What practices help control foaming or instability during wet-dispersion measurements? | Controlling stir speed, minimizing air entrainment, and adjusting surfactant levels were recommended. Consistent documentation of dispersion conditions and visual observation remain key to reproducibility. |
| 5. Should vendor software algorithms (e.g., smoothing or deconvolution) be standardized or independently verified for review? | Transparency in software settings and algorithm use is more critical than strict standardization. Regulators value full disclosure of processing parameters so reviewers can interpret how the reported distribution was derived. |

**Table S4.** Overview of Online Attendee Questions – Day 2 (Laser Diffraction Focus)

| **Category** | **Representative Question Topics** | **Number of Questions** | **General Trend or Interest** |
| --- | --- | --- | --- |
| **1. Sample Preparation and Dispersion Control** | Wet vs. dry dispersion; effect of stir speed and sonication; foaming and stability issues; managing agglomerates | **9** | Many participants focused on how dispersion conditions influence reproducibility. Practical issues—stirring rate, sonication energy, sample aging, and foaming—were recurring themes. |
| **2. Optical Parameters and Model Selection** | Refractive index accuracy; Mie vs. Fraunhofer models; absorption and scattering effects; choice of RI for multi-component systems | **6** | Strong interest in understanding how incorrect optical inputs alter particle-size output and how to justify chosen values in regulatory submissions. |
| **3. Method Validation and Transfer** | Repeatability across instruments; inter-lab variability; robustness testing; impact of operator technique | **5** | Attendees wanted guidance on how to design validation studies and define acceptable tolerances for LD methods used in comparative studies. |
| **4. Data Analysis and Software Algorithms** | Use of smoothing or deconvolution; reporting settings in submissions; cross-software comparability | **3** | Participants asked for clarity on how software parameters should be documented and whether vendors should standardize algorithms. |
| **5. Excipient Interference and Background Correction** | Truncation vs. placebo-background subtraction; validation of correction methods; matrix effects | **4** | This topic generated strong discussion. Questions reflected interest in how to quantify and report corrections for formulation interference. |
| **6. Regulatory and Comparability Issues** | Use of RLD lots to meet three-lot (“3 R”) expectation; harmonization of guidance; data transparency | **3** | Questions sought clarity on regulatory intent and how to demonstrate robust comparability when material availability is limited. |
| **7. Emerging Tools and Future Directions** | New optical or computational methods for error visualization; AI integration for pattern recognition | **2** | A smaller set of forward-looking questions addressed innovation and automation in LD data processing and quality control. |
